# Hysteria

**Published:** 1830-01-01

**Authors:** 


### ST. GEORGE'S HOSPITAL.

#### HYSTERIA.

There is not a more important practical lesson for a medical man to learn, than the power of distinguishing hysteria in its thousand forms from the various organic diseases which it apes. Unfortunately this is a power or a tact of which the generality of practitioners would seem to be destitute, for scarcely an admission day passes over our heads at this hospital without affording melancholy proofs of the evil consequences arising from mistakes of this nature. Young women are constantly applying with hysterical and nervous pains, whose complaints have been rivetted and constitutions shaken by injudicious and injurious depletion. We believe that the profession are beginning to discover that calomel and the lancet have been dealt around with a rather too liberal hand, and the various works on neuralgic diseases at present thronging from the press, give promise of a more rational appreciation of the affections of the nervous system. The following cases may not be devoid of utility in pointing out the frequency of hysterical pains in young women and the kind of treatment best adapted for their relief or removal. At the same time we must say that hysteria is too often an intractable complaint even under the most judicious system of management, and the supervention of mania, epilepsy, or even palsy shew, but too plainly, the tendency of diseases of this class to run into one another.

CASE 1. *Cough—Pain in the Side—Fluttering at the Heart—Mœnorrhagia.*

Mary Pearce, æt. 21, a servant unmarried, admitted July 24th, 1829, under the care of Dr. Chambers.

Dry cough excited on taking a full inspiration which also gives her pain in the left *hypochondrium*—fluttering at the heart—able to lie on either side—pulse quick but quite soft—skin moist—urine free and pale—no globus—nervousness approaching to hysteric passion—mænorrhagia every three weeks.

Has had pain on going up stairs, &c. for a month, but the present illness is only of a week's duration. It came on with cough, pain in the head and back, and shivering soon after her "period;" she knows no cause for her complaint.

*Empl. canth. lat. sinist. Mist. camph.* ℥iss. *Tr. hyosciam.* ʒss. *ter die. Diæta lactea.*

On the 24th being rather heated she was ordered house-physick and salines with antimony, and on the 27th, Ext. col. c. Ext. hyos. āā gr. v. every night. On the 31st the cough was almost entirely gone, the other symptoms had disappeared, and in a few days afterwards she was discharged cured.

CASE 2. *Chlorosis—pain in the Chest and Head.*

Cath. Cary, æt. 16, admitted May 21st under the care of Dr. Seymour.

Pains about the sternum increased on walking or standing, but not on making a deep inspiration—slight cough not worse at one time than another and unaccompanied with expectoration—pains over the head aggravated on rising in the mornings—occasional dimness of vision—no appetite for animal food which she says makes her sick—pulse languid—tongue moist and whitish—bowels rather confined—appearance extremely delicate and chlorotic—has never menstruated.

Ill between two and three months, having in the first instance caught a bad cold, accompanied with what seems to have been globus hystericus—applied at a dispensary where she was cured of the cold, &c. but became affected with the pains in the head and chest. Has attended at the dispensary with little relief to the present time. She says that when her cough was bad she occasionally spat blood.

*Tinct. ferr. amm.* ʒss. *Infus. cascar.* ʒx. *Tinct. cascar.* ʒj. *M. ter die. Pil. Al. c. Myrrh.* ℈ss. *altern. noct. Diæta media.*

On the 22d she was ordered the shower-bath every morning and a Burgundy pitch plaster was applied to the chest. On the 26th, the pain in the head was nearly gone and that in the chest much relieved, she was fatter, and had little cough. On the 4th of June she complained of more pain in the head but had none in the chest; she thought the bath agreed with her. On the 3d of July she was sufficiently well to leave the house. At this time she was infinitely better than she had been on her admission, but could scarcely be said to be cured, as the chlorotic state still continued. The cough was gone, the pains were very nearly so.

CASE III. *Pains in Head, Chest, &c. —Palpitations—Scanty Menstruation.*

Sarah Byatt, æt. 22, admitted June 10th, 1829, under the care of Dr. Seymour.

Pains in the head, right and sometimes left side of chest down to the ossa ilii, aggravated on inspiration or by pressure, but out of all proportion to the force employed—palpitations—flatulence—borborygmi—heart-burn—bowels confined without medicine—catamenia scanty—appearance hysterical. Has been ill ten months, bled, leeched, and purged with the effect of aggravating her complaint. Blisters afforded her some relief.

℞. *Tinct. valer. amm.* ʒj.—*Mist. camph.* ʒx. *M. ter die. Baln. imbrif. o. m.*

She continued much the same till the 19th, when she still complained of pain in the right side and shoulder, and the tongue was rather white. The medicine was altered to,—

*Tr. castor.* ʒss.—*Aq. cinn., Aq. font.* āā ʒv. *M. bis die.*

On the 22d her catamenia being on her, the medicines were omitted, but on the 25th they were resumed. On the 28th there was pain in the right side of the chest, shoulder, and hypochondrium, and the pulse was quick and feeble. The mist. ferr. c. was ordered bis die. On the 30th, in consequence of the sluggish state of bowels, senna draught was directed to be taken twice a week, and on the 17th of July, in consequence of the severity of the pain about the right hypochondriac and iliac regions, a blister was applied. On addressing her there was nervousness observed almost approaching to chorea. On the 21st, the catamenia again appeared, and the medicines were omitted. On the 24th she looked very well, but still complained of the pain in the right *iliac* region increased on inspiration, &c. and evidently disliked resuming the use of the shower-bath. In the course of a few days she was dismissed, having little of consequence the matter with her.

Case IV. *Pain of Chest—Severe Cough —Scanty Menstruation.*

Maria Anson, æt. 18, a florid country girl, admitted Sept. 10th, 1829, under the care of Dr. Chambers.

Pain just under the xiphoid cartilage, appearing night and morning, and aggravated on full inspiration—no pain elsewhere—head-ache *when* the pain in the chest is present—severe cough at night with little expectoration—shortness of breath—borborygmi—no globus —pulse rather sharp and between 70 and 80—face flushed and hysteric looking—skin warm and moist—tongue clean—bowels open—urine free—catamenia at present on her, but scantily.

Ill two years with the present symptoms sometimes better and sometimes worse. Three months ago had pain in the "right side," (region of colon) for which she was bled, &c. with little benefit. Catamenia appeared at the age of 14, and have continued with regularity till the last three months, during which time they have not made their appearance till at present. Has been leeched, bled, blistered, &c. without relief; father died of a decline.

*V.S. ad* ℥xij.—*Inf. ros.* ℥iss.—*Mag. sulph.* ʒij.—*Spir. æth. sulph. c.* ʒss. *ter die. Diæta lactea.*

On the 11th the cough being very troublesome at night, she was ordered an opiate at bed-time, and on the 16th a blister was applied to the chest. On the 18th the former medicines were discontinued and pil. galb. c. ℈ss. given thrice daily. On the 25th she was ordered a shower-bath every morning. On the 3d of October, after exposure to cold, she was attacked with slight inflammation of the lungs, which was immediately treated and relieved by a small bleeding, salines with antimony and hyosciamus, and a brisk cathartic of calomel, ipecacuanha, and jalap. On the 10th she complained of pain over the right eye at night (the clavus hystericus) pulse soft and frequent, skin cool and moist, tongue rather red, bowels open, urine free. Camphor mixture with the spiritus ammoniæ fœtidus was prescribed, and on the 12th the patient was discharged cured.

In all these cases, except that of Byatt, the prominent symptoms were pain in the chest and cough, the latter being extremely severe in the last case, that of Anson. All the patients presented unequivocal marks of the hysteric diathesis, if we may make use of such a term, viz. derangement of the catamenial secretion, flatulence, costiveness, borborygmi, globus, or clavus hystericus. In the second case, that of Carey, the general appearance and thoracic symptoms might have led many persons to imagine that phthisis was present, and probably had she been blistered and leeched and bled they would have contributed to the correctness of their own diagnosis, and "made the food they fed on." In the last case, that of Anson, the pain in the chest, the shortness of breath and the cough, together with the circumstance of her father having died of a decline according to her own report, might likewise have induced many to treat her for inflammation in the lungs, as indeed she had been treated without benefit, prior to her admission. The general appearance, however, the clavus hystericus, and the flatulence sufficiently pointed out the hysterical character of the case, and it subsequently turned out that two or three months previously her sweetheart had given her a gonorrhœa, for which she indignantly cast him off, and from which period her more aggravated symptoms commenced. At the same time it must be allowed that this was in some degree a mixed case, the catarrhal affection being more than usually severe, and justifying, if not strictly requiring, moderate antiphlogistic measures. These cases are the ones that demand most skill on the part of the medical man, and consequently are those which are most frequently mistreated.

For our own parts, we believe that there are very few cases of this kind that require general depletion, very many indeed that are aggravated and rendered almost incurable by its adoption. If depletion is necessary it should be local by means of leeches and cupping, but counter-irriration by blisters or the tartar-emetic is to be preferred. In almost every instance the patient experiences relief from blisters, but there is this disadvantage attending their use, that it directs the patient's attention to the *part*, whereas it should be the object of the practitioner to draw it off and divert it. In a future report we will detail some more aggravated cases of hysteria, and also some where the heart was the principal organ affected.

I. EXCISION OF THE ELBOW-JOINT.

